# Single acquisition label-free histology-like imaging with dual-contrast photoacoustic remote sensing microscopy

**DOI:** 10.1117/1.JBO.26.5.056007

**Published:** 2021-05-25

**Authors:** Benjamin Ecclestone, Deepak Dinakaran, Parsin Haji Reza

**Affiliations:** a University of Waterloo, Faculty of Engineering, Systems Design Engineering, PhotoMedicine Labs, Waterloo, Ontario, Canada; b illumiSonics, Waterloo, Canada; c University of Alberta, Department of Oncology, Edmonton, Alberta, Canada

**Keywords:** photoacoustic remote sensing, photoacoustic, histology, optical imaging, label-free histology

## Abstract

**Significance:** Histopathological analysis of tissues is an essential tool for grading, staging, diagnosing, and resecting cancers and other malignancies. Current histopathological imaging techniques require substantial sample processing, prior to staining with hematoxylin and eosin (H&E) dyes, to highlight nuclear and cellular morphology. Sample preparation and staining is resource intensive and introduces potential for variability during sample preparation.

**Aim:** We present a method for direct label-free histopathological assessment of formalin-fixed paraffin-embedded tissue blocks and thin tissue sections using a dual-contrast photoacoustic remote sensing (PARS) microscopy system.

**Approach:** To emulate the nuclear and cellular contrast of H&E staining, we leverage unique properties of the PARS system. Here, the ultraviolet excitation PARS microscope takes advantage of DNA’s unique optical absorption to provide nuclear contrast analogous to hematoxylin staining of cell nuclei. Concurrently, the optical scattering contrast of the PARS detection system is leveraged to provide bulk tissue contrast reminiscent of eosin staining of cell membranes.

**Results:** We demonstrate the efficacy of this technique by imaging human breast tissue and human skin tissues in formalin-fixed paraffin-embedded tissue blocks and frozen sections, respectively. Salient nuclear and extranuclear features such as cancerous cells, glands and ducts, adipocytes, and stromal structures such as collagen are captured.

**Conclusions:** The presented dual-contrast PARS microscope enables label-free visualization of tissues with contrast and quality comparable to the current gold standard for histopathological analysis. Thus, the proposed system is well positioned to augment existing histopathological workflows, providing histological imaging directly on unstained tissue blocks and sections.

## 1 Introduction

Histopathological analysis of tissues is an essential tool in the diagnosis and treatment of cancers and other malignancies. Histopathology is performed intraoperatively or for diagnostics to provide visualizations of nuclear structures and microscopic tissue morphology. This enables identification of tissue type, and differentiation of pathological or cancerous from healthy tissue and necrotic from living tissues. Intraoperatively, histopathology helps to guide surgeons in excising cancerous regions while removing only a minimal margin of healthy tissue, thereby preserving the organ’s function to the greatest extent.^1^ Outside the operating room, histopathological analysis aids in decisions on adjunct treatments, as well as diagnosing, grading, and staging malignancies. The gold standard for performing histopathological imaging is formalin-fixed paraffin embedded (FFPE) preparation followed by hematoxylin and eosin (H&E) staining.^2^ However, frozen preparation methods have become a more common technique in recent years.^3^ Though the exact processing differs significantly, the basis of the frozen and paraffin methods is equivalent. First, excised surgical tissues or biopsies are deposited into a substrate forming a solid tissue block.^2^^,^^3^ Once embedded, thin sections of tissue are shaved from the block using a microtome. These thin sections are then fixed to a microscope slide and finally stained with immunohistochemical dye, usually H&E. Specifically, hematoxylin dye targets nuclei, while eosin targets cell cytoplasm, collagen, and a few other extracellular structures.^2^ While this technique provides excellent visualization compatible with conventional transmission mode light microscopy, sectioning and staining tissues is an involved and resource intensive process. Producing thin sections from tissue blocks requires high precision, sometimes necessitating multiple attempts to recover an adequate slice.^3^ Once collected, sections must be fixed to a slide and stained, a process that is subject to some variability, potentially contributing to imaging artifacts.^3^^,^^4^ This time-consuming procedure can delay diagnosis by days or even weeks in some instances.^5^ In the case of frozen preparations, staining and sectioning accounts for over one-third of the total preparation time.^6^ These process limitations may result in nonideal clinical practices and poorer patient outcomes.

In the ideal case, contrast similar to that provided by H&E staining could be acquired label-free directly from unstained tissue preparations. By extension, such a technique would not modify samples during imaging, preserving tissues for further immunohistochemical analysis. For maximal utility, this ideal system should not be limited to a single sample format but should perform on a variety of preparations, such as FFPE tissue blocks, FFPE tissue sections, and frozen sections. Visualizing tissue morphology directly on preserved samples would mitigate sectioning and staining requirements, saving time and resources. Thus far, several pragmatic hurdles have prevented direct histology-like imaging of unstained FFPE and frozen preparations. While thin sections are translucent and therefore compatible with transmission mode imaging, FFPE blocks are typically a few millimeters thick and opaque. Thus, the sample morphology necessitates a reflection mode modality. Moreover, capturing high fidelity nuclear and cellular features without exogenous contrast agents represents a substantial hurdle. The most popular techniques such as fluorescence microscopy,^7^ microscopy with ultraviolet (UV) surface excitation,^8^ and light sheet microscopy^9^ require exogenous dyes. Despite these challenges, a few modalities have shown histology-like imaging capabilities in tissue preparations. Stimulated Raman scattering has produced promising label-free histological-like results.^10^ However, this has not been shown in FFPE tissue blocks. Conversely, optical coherence tomography (OCT) has shown label-free imaging capabilities in a variety of sample formats.^11^ However, the optical scattering contrast of OCT does not readily provide chromophore-specific visualization similar to H&E processing.^11^ One alternative technique, optical resolution photoacoustic imaging provides highly chromophore-specific contrast.^12^ Recently, it has been applied to histological imaging in a variety of preserved samples.^12^ This technique leverages the photoacoustic effect to capture the intrinsic optical absorption contrast of tissues. In photoacoustic imaging, a pulsed excitation laser is used to deposit localized optical energy into a sample, which then undergoes thermoelastic expansion.^12^ The localized thermoelastic expansion results in an outward propagating acoustic pressure wave, which is traditionally detected with an acoustically coupled ultrasound transducer.^12^ Unfortunately, the requirement for acoustic detection presents several challenges. Acoustic transducers are typically bulky, have known interoperator technique reliability issues, and sometimes require immersion in a coupling media such as water to function.^13^

Recently, photoacoustic remote sensing (PARS) microscopy has emerged as an all-optical noncontact alternative to traditional optical resolution photoacoustic microscopy. PARS replaces the acoustically coupled ultrasound transducer with a detection laser.^14^ Photoacoustic signals are then detected as pressure-induced modulations in the backscattered magnitude of the detection beam.^14^ Observing backscattering in a reflection mode architecture allows PARS to image thick samples.^14^ Moreover, PARS may provide chromophore-specific contrast by selecting excitation wavelengths to target unique biomolecule absorption spectra.^15^^–^^18^ Applied to histological imaging, PARS has successfully used UV excitation to capture nuclear structures.^15^^–^^21^ This technique is efficacious in providing hematoxylin-like contrast in frozen sections and paraffin-embedded blocks and sections.^15^^–^^21^ However, these UV-PARS visualizations lack some essential diagnostic details. To provide the full picture, bulk tissue contrast analogous to eosin staining of cell membranes is required to provide structure and reference for the visualized nuclei. This is of particular relevance in tissues with low nuclear density, such as adipose, where the adipocytes are made up of sparse nuclei surrounded by largely lipid-containing vacuoles in the cytoplasm. Previously, PARS has provided complete H&E emulation using a tunable excitation source to independently target the absorption peaks of DNA and cell membrane structures.^17^^,^^18^ While effective in both thin sections and tissue blocks, this technique was largely limited in field of view, resolution, and imaging speed since it required the use of a slow (1 kHz) tunable excitation source.^17^^,^^18^ In this paper, we propose leveraging the optical scattering microscope architecture of the PARS detection system to provide bulk tissue contrast, while using an UV excitation to capture nuclear structures. For the first time, we use the unique ability of PARS microscopy to concurrently provide both label-free absorption and scattering contrast. To the extent of our knowledge, PARS is the only optical imaging technique able to provide dual-contrast optical scattering and optical absorption simultaneously. We capture eosin-like visualizations by imaging the disparity in the optical scattering of the tissue substrate and embedded tumor tissue. Concurrently, the UV excitation is used to highlight nuclear morphology, analogous to hematoxylin staining. Combining the PARS optical scattering and absorption images, we provide emulated H&E-like visualizations in a single acquisition. With the proposed technique, images are captured substantially faster than equivalent visualizations made with our previously reported multiwavelength system. We demonstrate the proposed method in thin human tissue sections and FFPE blocks. Employed in a clinical setting, the proposed technique would allow H&E-like images to be captured label-free in embedded tissue samples. Thus, PARS is well positioned to supplement existing tissue analysis techniques, potentially mitigating the requirement for sectioning and staining tissues prior to analysis.

## 2 Methods

### 2.1 System Architecture

**Fig. 1:**
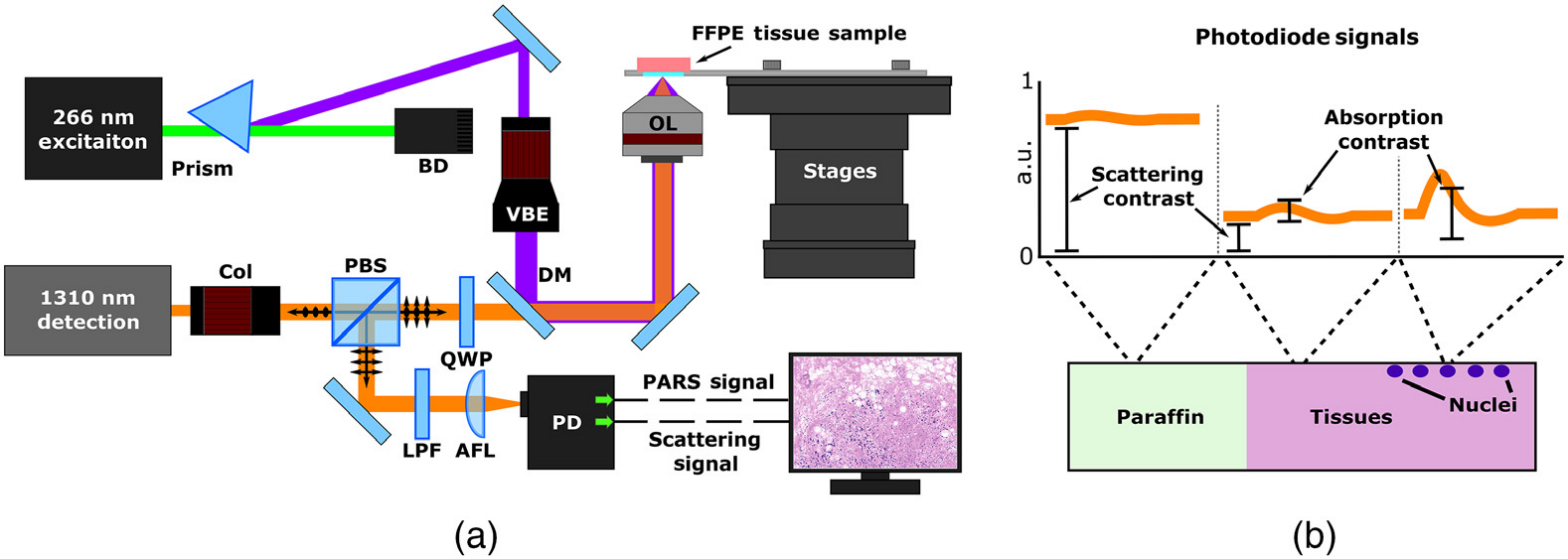
Simplified experimental system setup. (a) Component labels are as follows: dichroic mirror, DM; variable beam expander, VBE; beam dump, BD; objective lens, OL; collimator, Col; quarter waveplate, QWP; polarized beam splitter, PBS; long-pass filter, LPF; aspheric focal lens, AFL; and photodiode, PD. (b) PARS absorption and scattering signals encoded in the backreflected detection light. PARS scattering is captured by the lower frequency backscattering intensity. PARS absorption contrast is contained within the nanosecond-scale photoacoustic pressure induced backreflected intensity modulations.

The architecture of the experimental system is illustrated in Fig. 1(a). The UV PARS excitation is provided by a 266 nm, 50-kHz, 500-ps pulsed laser (WEDGE XF 266, Bright Solutions). The 266-nm excitation beam is first separated from the residual 532 nm output, using a prism (PS862, ThorLabs, Inc.). Once isolated, the excitation beam is expanded (BE05-266, ThorLabs, Inc.) and combined with the detection via a dichroic mirror (HBSY234, ThorLabs, Inc.). The PARS detection and optical scattering source is a 1310-nm continuous wave superluminescent diode (S5FC1018P, ThorLabs, Inc.). Collimated detection light is passed through a polarizing beam splitter and quarter wave plate and then combined with the excitation. Once combined, the beams are cofocused onto the sample with a 0.5-NA reflective objective (LMM-15X-UVV, ThorLabs, Inc.). Backscattered detection light returns to the quarter wave plate by the same path as forward propagation. Upon return, the polarizing beam splitter redirects the detection beam through spatial and chromatic filters toward the photodiode where the PARS signals are captured.

### 2.2 Image Formation

In this architecture, two separate outputs are collected from the photodetector. The first is an unamplified and unfiltered output, corresponding to the PARS scattering contrast attributed to the 1310-nm detection source [Fig. 1(b), scattering contrast]. This signal is contained within the low frequency (near DC) content of the optical signals captured by the photodiode [Fig. 1(b), scattering contrast]. The second signal is a band-pass filtered and amplified output, which isolates nanosecond-scale modulation in the optical scattering [Fig. 1(b), absorption contrast]. This signal is the characteristic PARS absorption contrast. The two signals are captured simultaneously using a multichannel high-speed digitizer (RZE-004-300, Gage Applied). To form a complete frame, the mechanical stages are used to raster scan the sample across the imaging head. When scanning, the excitation is pulsed at 50 kHz, while the velocity of the stages is modified to achieve the desired lateral spacing between interrogation points. At each excitation pulse, the PARS scattering amplitude, absorption amplitude and location are recorded. To reconstruct the PARS absorption and scattering images, the raw data are imposed onto a Cartesian grid based on the location signal at each interrogation. Color mapping analogous to H&E staining is then applied to the PARS absorption and scattering images, respectively. To form the final emulated H&E image, a basic linear mixing is performed, combining the contrast of the absorption and scattering frames.

### 2.3 Sample Preparation

Two different formats of human tissue samples were used in this study. Thin frozen sections of human skin tissues were collected from Mohs surgeries, and FFPE breast tissue blocks were recovered from breast conserving surgery. Tissue samples used during this study were acquired under protocols approved by the Research Ethics Board of Alberta (HREBA.CC-18-0277) and the University of Waterloo Health Research Ethics Committee (Humans 40275). During this study, patient consent was waived as samples were archival tissue not required for diagnostic purposes, and no patient identifiers were provided to the researchers.

#### 2.3.1 Frozen tissue sections

Collection and processing of thin frozen skin sections was performed as follows. Tissues samples were selected from Mohs surgical resections. Chosen specimens were embedded in a cryostat substrate and cooled to ∼−25°C, forming an embedded frozen tissue block. Several 5- to 10-μm thick sections were cut from the tissue blocks with a cryotome. Resulting thin sections were fixed to slides and dried at 55°C for 1 min. The fixed and unstained tissue slices were then transported to the PhotoMedicine Labs and imaged with the PARS microscope. Following PARS imaging, the unstained slides were returned to the clinicians to undergo standard H&E staining. Here, slides were stained with H&E dyes and then covered with mounting media and a cover slip. The resulting fixed H&E slides were imaged with a transmission mode brightfield microscope (Zeiss Axioscope 2 with Zeiss Axiocam HR).

#### 2.3.2 Formalin-fixed paraffin-embedded tissue block

FFPE human breast tissues were collected and processed as follows. Breast tissue samples were selected during breast conserving surgery. Selected tissues were placed into a 10% formalin fixative solution within 20 min of extraction. After 24 h of fixation, tissues samples were embedded into a paraffin wax substrate forming FFPE tissue blocks. Several ∼5  μm sections were cut from the surface of the blocks using a microtome. The resulting thin sections were fixed to slides and stained with H&E dye. Stained slides were completed by covering tissues with mounting media and a cover slip. One nondiagnostic section was set aside from the set of prepared H&E slides to provide a comparison between H&E techniques and PARS imaging of tissue blocks. Following processing, the tissue block was transported to the PhotoMedicine Labs and imaged with the PARS microscope. The corresponding H&E slide was imaged with a standard brightfield microscope (Zeiss Axioscope 2 with Zeiss Axiocam HR).

## 3 Results and Discussion

In principle, the proposed dual-contrast PARS system expands upon previously reported PARS histological imaging techniques. Most recent works feature the use of UV excitation to target the absorption contrast of nuclei.^15^^–^^21^ These techniques have subsequently provided hematoxylin-like visualizations of nuclear structures in tissue blocks, slides, and fresh tissues.^15^^–^^21^ However, these techniques do not provide full H&E emulation, as they lack the extranuclear tissue structures captured by eosin staining. Here, we propose approaching this problem by leveraging, for the first time, PARS unique dual scattering and absorption contrast. In PARS systems, a cofocused pulsed excitation and continuous wave detection laser pair are used to capture photoacoustic absorption contrast.^14^ The excitation induces photoacoustic signals by depositing focused pulses of optical energy into the sample.^14^ The absorption contrast is then captured as nanosecond scale pressure-induced modulations in the backscattered intensity of the cofocused detection laser.^14^ Usually, to capture MHz-scale PARS modulations, the time-resolved backscattering magnitude is band pass filtered to isolate the absorption signals.^14^ However, in this embodiment, we also capture low frequency trends in the backscattering signals, indicative of the samples local scattering properties. Therefore, the wavelength of the detection source may then be chosen to target sample-specific scattering contrast. Here, the detection wavelength is selected to target differences in the optical properties of the tissue and the surrounding paraffin or frozen embedding substrate. In this case, the 1310-nm detection is scattered more by the paraffin wax and frozen substrates compared with the tissues. As a result, the tissues appear as darker regions within the PARS scattering visualizations. We then highlight predominately tissue morphology, analogous to eosin staining, by applying an inverted colormap to the PARS scattering images. This method can be augmented into most PARS systems since the proposed technique leverages the scattering contrast inherently provided by the PARS detection. The most important step is independently extracting the absorption and scattering signals. Once extracted, the scattering and absorption images may be formed independently and then combined with a basic linear mixing technique.

**Fig. 2:**
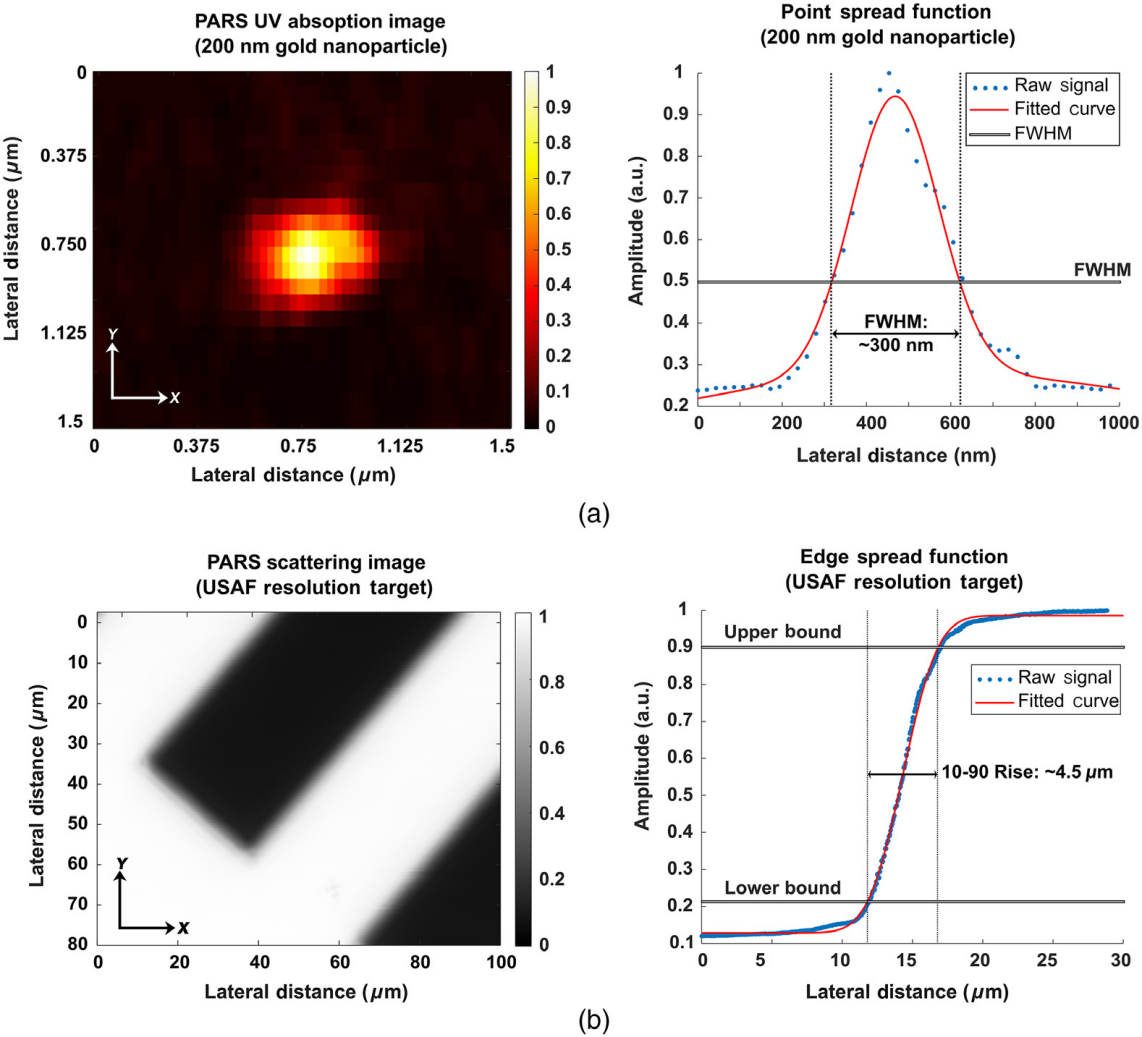
Characterization of the PARS absorption and scattering resolutions. (a) PARS 266-nm absorption contrast image of a 200-nm diameter gold nanoparticle. This image is acquired with a ∼20-nm lateral step size in the x dimension and a 50-nm lateral step size in the y dimension. The corresponding point spread function collected from the nanoparticle is shown to the right, revealing an FWHM resolution of ∼300  nm. (b) PARS 1310 nm scattering image from a USAF resolution target. This image is collected with a 100-nm lateral step size in the x dimension and a 100-nm lateral step size in the y dimension. The corresponding edge spread function collected from the USAF target is shown to the right, revealing a 10-90 rise resolution of ∼4.5  μm.

**Fig. 3:**
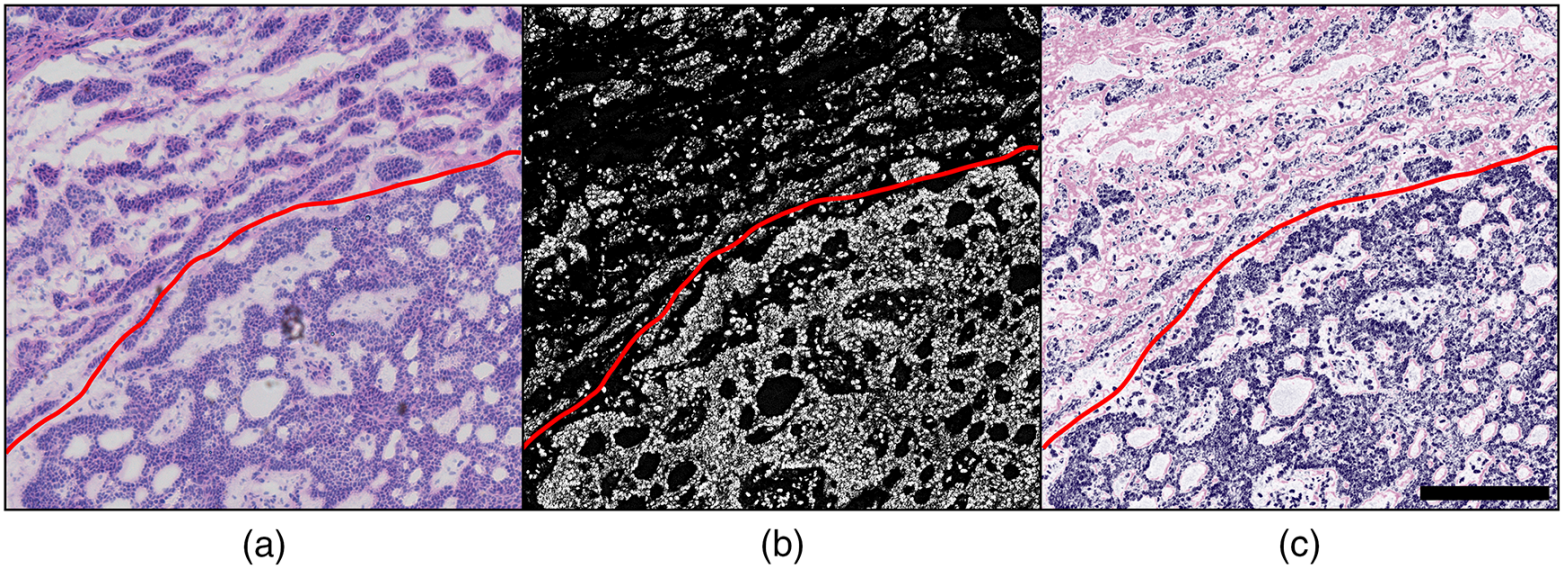
One-to-one comparison of brightfield H&E imaging, direct PARS, and the proposed PARS absorption and scattering technique in human skin tissue with BCC. PARS images are 1 by 1 mm, captured with 4  pixels/μm sampling, 16 megapixels total. (a) Brightfield image of H&E-stained tissue with BCC demonstrating the border of invasive cancer (bottom of red border) versus normal tissue (top of red border). (b) PARS image of the same unstained sample; red border denotes the cancer boundary. (c) Combined PARS absorption and scattering image of the same unstained sample; red border denotes the cancer boundary. Scale bar: 250  μm.

**Fig. 4:**
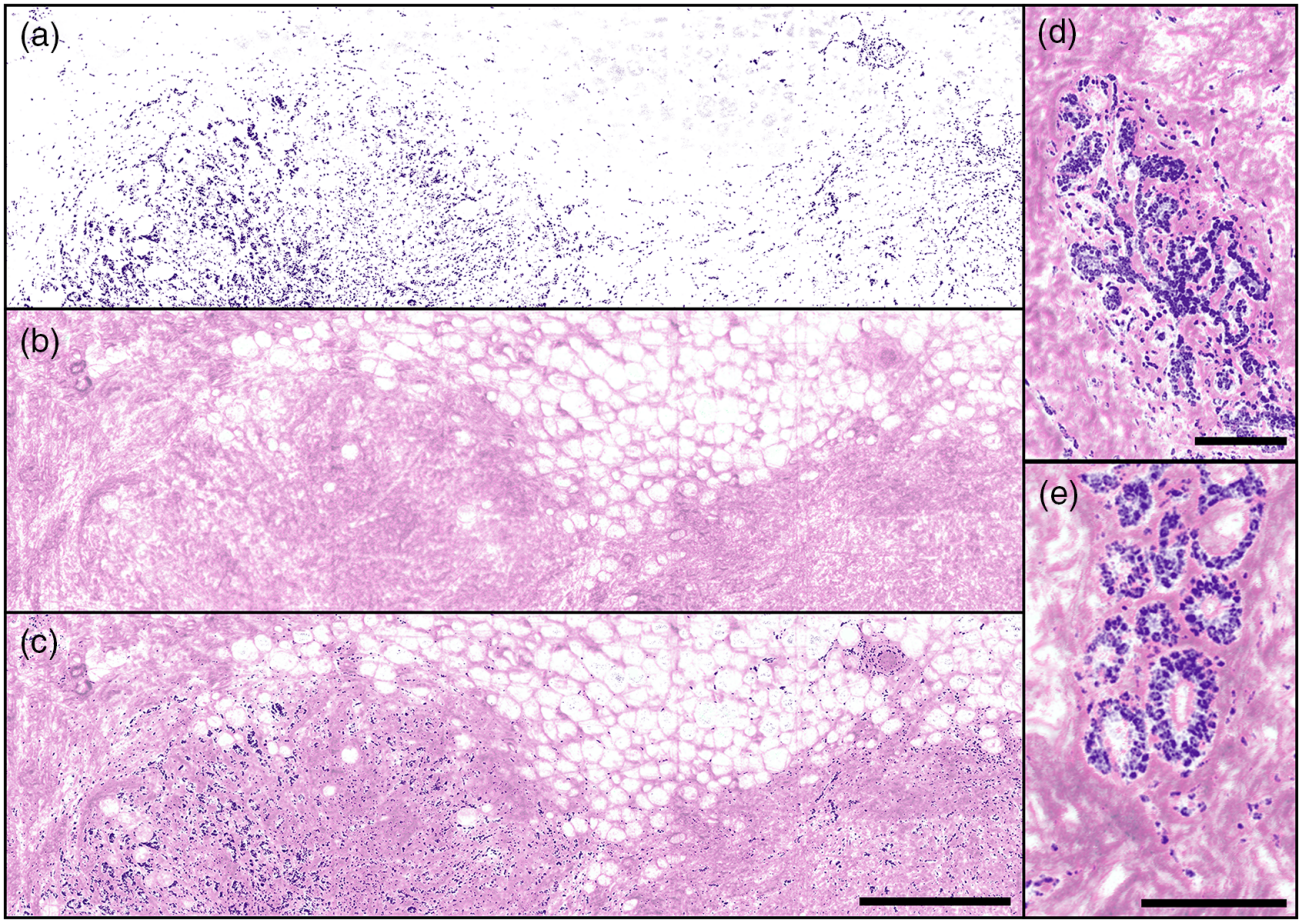
Individual and combined PARS absorption and scattering images of FFPE human breast tissues in block form, providing emulated H&E stain contrast. PARS images captured with 2  pixels/μm sampling. (a) UV-PARS image of nuclei providing contrast analogous to hematoxylin staining of cell nuclei. The distribution of nuclei in the breast tissue specimen is evident but by itself is not diagnostic. (b) PARS scattering image providing visualizations analogous to eosin staining of cell membranes. This image reveals regions of adipose (clear voids) and dense, collagen-rich stromal tissue (pink-colored) and the organization of these structures in relation to each other. (c) Combined [(a) and (b)] emulated H&E PARS image of normal postmenopausal breast tissue with sparse and atrophic glands with higher nuclear densities (bottom left and right) with mainly adipose (top) and connective tissue (false-colored pink) comprising the majority of the sample. Scale bar: 500  μm. Images are ∼12  megapixels. (d), (e) Combined emulated H&E PARS images of normal postmenopausal breast tissue with atrophic glands surrounded by connective tissue. These smaller images are independent regions, not located within (a)–(c). (d) Scale bar: 100  μm. Image is ∼0.4  megapixels. (e) Scale bar: 125  μm. Image is ∼0.3  megapixels.

**Fig. 5:**
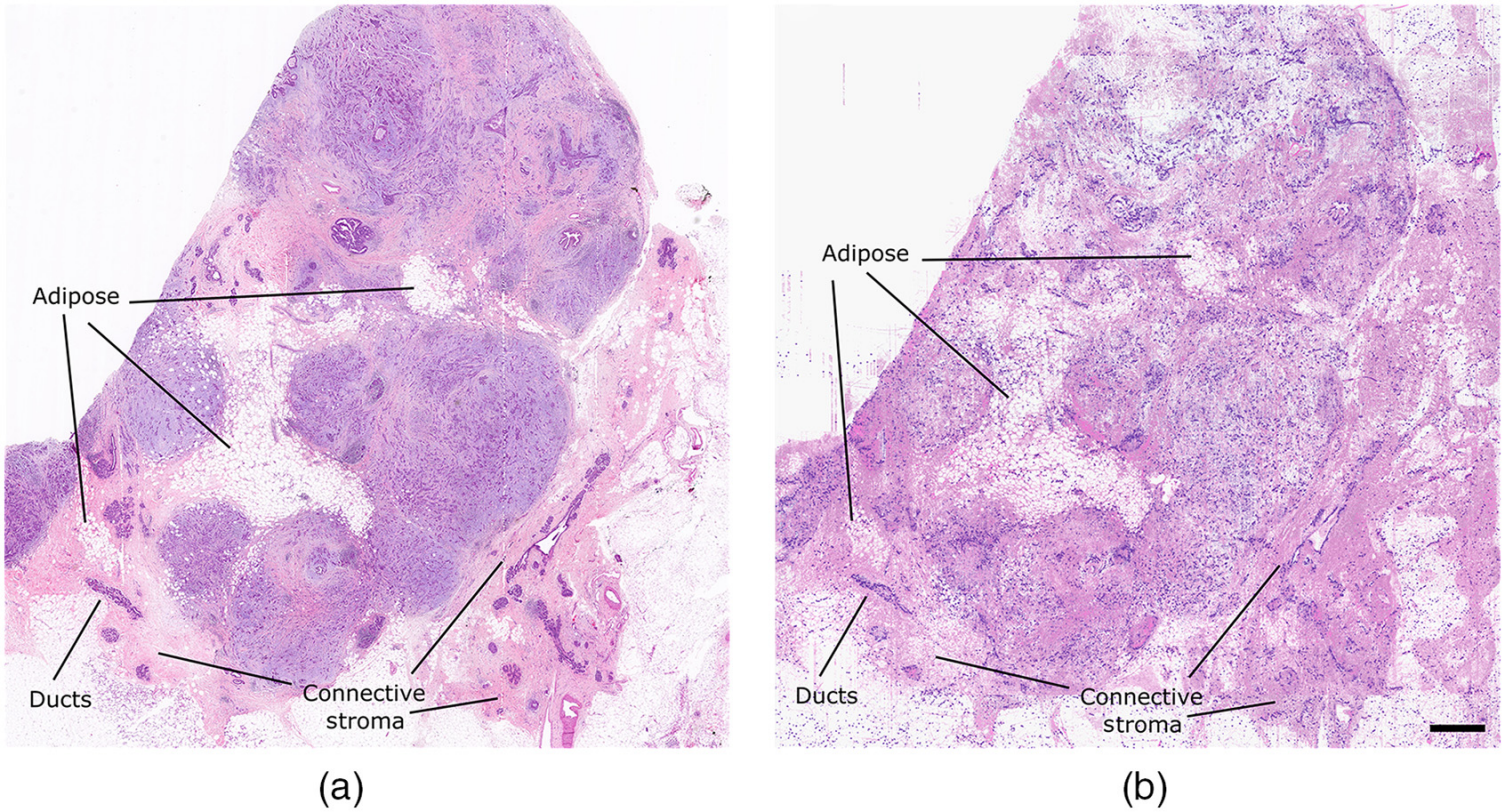
Comparison of brightfield H&E imaging and the proposed dual-contrast PARS absorption and scattering technique in FFPE human breast tissues in block form. PARS image is 13.25 by 14 mm, with 0.25  pixels/μm sampling, ∼12  megapixels total. (a) Brightfield image of H&E-stained FFPE tissues sectioned from an FFPE tissue block. (b) Dual-contrast PARS image from the surface of the FFPE human breast tissue block. Scale bar: 1 mm.

In the presented architecture, we utilize a 0.5-NA reflective objective lens, with a 1310-nm detection and a 266-nm excitation. The UV excitation provides ∼300-nm lateral and ∼1.4-μm axial resolution absorption contrast [Fig. 2(a)]. This was determined based on the full width half maximum (FWHM) of the point spread function generated from imaging 200-nm-diameter gold nanoparticles. The detection provides ∼4.5-μm lateral and ∼68-μm axial resolution scattering contrast [Fig. 2(b)]. This was determined from the 10% to 90% response rise of an edge spread function generated on a USAF resolution target. While the lateral resolution defines the sharpness of the images, the axial resolution provides depth discrimination within samples. In thick tissues, we leverage the optical sectioning of the detection and excitation to capture only a thin section of scattering and absorption contrast. While imaging such specimens, the continuous wave detection operates at ∼2  mW, while the UV excitation uses ∼0.8  nJ pulses, at a rate of 50 kHz. In the current embodiment, the 50-kHz repetition rate of the excitation source limits imaging speeds. Acquiring the scattering images does not affect the acquisition rate since the scattering and absorption images are acquired simultaneously. Therefore, this system provides H&E emulation with imaging rates comparable to previously reported UV excitation PARS systems.^21^ The current implementation of the system is capable of collecting high-resolution images with 250-nm spatial sampling at a rate of $∼6\;\min/{\mathrm{mm}}^{2}$ (∼6  min for an area equivalent to that shown in Fig. 3). Concurrently, 500-nm spatial sampling scans may be captured at a rate of $∼90\;s/{\mathrm{mm}}^{2}$ (∼5  min for an area equivalent to that shown in Fig. 4). Finally, large grossing scans with 4-μm spatial sampling are captured at a rate of $5\;\min/{\mathrm{cm}}^{2}$ (about ∼12  min for the ∼14-mm scan shown in Fig. 5).

We first apply the proposed technique to imaging unstained thin tissue sections. Here, human skin tissues provide a one-to-one comparison between the proposed technique, UV PARS imaging, and brightfield H&E preparation (Fig. 3). These samples were previously used to provide the first direct comparison between PARS and H&E imaging.^21^ As outlined in the corresponding publication, unstained frozen skin tissue sections were collected and scanned with the PARS microscope.^21^ Following PARS imaging, the unstained sections were stained and imaged with a standard light microscope.^21^ The corresponding H&E and PARS visualizations of a small region of this tissue are shown in Figs. 3(a) and 3(b), respectively. This high-resolution close-up of a tissue sample with basal cell carcinoma (BCC) shows a characteristic invasive tumor front, made up of unorganized sheets of cells with aberrant, atypical nuclei. This particular field-of-view captures the clinically relevant boundary between healthy tissue and dense tumor tissue, which is otherwise known as the tumor margin (red line). Visualizing this margin allows clinicians to determine if complete resection of the tumor is achieved. The PARS image accurately captures the nuclear structure in this region [Fig. 3(b)], providing a one-to-one match between individual nuclei within the H&E image [Fig. 3(a)]. While this contrast successfully highlights features including the tumor margin and localized nuclear atypia, the result is equivalent to using only hematoxylin stain and thus is not as informative as traditional H&E. In contrast to the nuclear dense tumor, the nonnuclear morphology of the healthy tissue in the upper left of the image is poorly represented [Fig. 3(b)]. To remedy this, we apply the proposed technique to obtain eosin-like visualizations. We mix the PARS scattering contrast of the 1310-nm detection with the PARS UV absorption image. The resulting emulated PARS H&E image is shown in Fig. 3(c) (this 1 mm by 1 mm, 16-megapixel visualization is captured with 250-nm spatial sampling in ∼6  min). In contrast to the pure PARS UV absorption results, the emulated H&E visualization highlights nearly identical structures to that of the stained slide. However, there are some subtle differences in the visualizations as the H&E preparation exhibits nonideal eosin staining contrast. Some common causes of this artifact include too high of a pH during eosin staining, a too diluted eosin stain, too short of an eosin staining time, or using too thin of a frozen tissue section. As a result, the extranuclear tissue features captured by the PARS system appear more saturated than those presented in the H&E image. However, the extranuclear structures of the nuclear sparse tissue in the upper left of the image are clearly visible in both the dual-contrast PARS and the H&E images. In nuclear-sparse areas, the PARS scattering contrast allows connective tissue stroma to be distinguished from voids of likely adipose tissue. With the full context of the simulated H&E staining, this slide can be interpreted as the tumor margin (bottom right) invading into regions of subcutaneous tissue. Applied in a clinical setting, directly imaging preserved tissues with this technique could enable such interpretations without the need for staining.

Applied in FFPE tissue blocks, this technique successfully recovers both nuclear and bulk tissue contrast (Fig. 4). The PARS image capturing nuclear structures with hematoxylin-like contrast is shown in Fig. 4(a). In isolation, this provides clinically relevant features including internuclear density, cross-section, morphology, and internuclear spacing. However, there is a distinct lack of extranuclear tissue morphology. To provide the missing structural details, we therefore leverage the optical scattering contrast of the PARS detection [Fig. 4(b)]. Based on the difference in scattering properties between the sample and the paraffin substrate, we capture eosin-like visualizations within the FFPE tissue block. In this case, the scattering image reveals a large region of adipose tissue in the upper right and a denser region of tissue in the lower left. While these details are of some clinical relevance, without the underlying nuclear structures, the regions of tissue are nondiagnostic. Therefore, we provide a fully emulated H&E image with nuclear and extranuclear contrast. To do so, the PARS scattering and absorption images are combined using a basic linear mixing technique. The resulting fully emulated H&E image, providing combined nuclear and bulk tissue structures, is shown in [Fig. 4(c)]. (This 12-megapixel visualization is captured with 500-nm spatial sampling at a rate of $∼1.5\;\min/{\mathrm{mm}}^{2}$.) With the full context of the simulated H&E staining, we can identify further clinical details such as the connective tissue stroma, made up mainly of eosin-stained collagen fibers. The connective tissue stroma can also now be distinguished from interspersed vacuoles, which, in FFPE samples such as this, most often represent adipose tissue. Observing smaller regions of the FFPE breast tissue with the PARS-emulated H&E visualization [Figs. 4(d) and 4(e)], the morphology of mammary glands and ducts is clearly visible. The small field images, Figs. 4(d) and 4(e), are independent regions of tissue from that presented in Figs. 4(a)–4(c). These enhanced regions reveal normal postmenopausal breast tissue with sparse and atrophic glands surrounded by connective tissue. With the information gathered about the nuclear (hematoxylin staining) structures and connective tissue (eosin staining), this tissue block is clearly identifiable as a normal postmenopausal breast tissue specimen. We emphasize that these PARS images are captured directly on an FFPE tissue block not a thin tissue section. This is the first technique to provide single acquisition H&E-like visualizations in such FFPE tissue blocks.

While the previous visualizations featured high-resolution small field images, the proposed technique is not limited to this application. This method is also applied to large area scanning in FFPE tissue blocks (Fig. 5). Here, we capture an entire FFPE tissue specimen, with 4-μm spatial sampling (∼12-megapixel image) in ∼12  min. In clinical settings, rapid previews of large sections of tissues are crucial for histological analysis. Clinicians may use grossing scans to select clinically relevant regions, which should be further assessed with higher resolution imaging. With the proposed technique, these grossing visualizations (Fig. 5) provide nuclear and bulk tissue contrast. Thus, we provide rapid assessment of generalized tissue structures, tissue type, density, and organization. Moreover, bulk nuclear morphology and internuclear spacing can be assessed simultaneously. We emphasize that the large field dual-contrast PARS visualization [Fig. 5(b)] is captured directly from the FFPE tissue block surface without any sample preparation. In this case, the dual-contrast PARS image reveals several pockets of adipose tissue, dense regions of stromal tissues, and glandular and ductal structures. A comparable region of tissue captured within an H&E-stained tissue section, cut from the same face of the tissue block, is shown in Fig. 5(a). The H&E visualization highlights analogous regions of connective tissue stroma with interspersed pockets of adipose and similarly distributed glandular and ductal structures. However, while the general morphology of the H&E and PARS samples is the same, the H&E image is not directly comparable to the PARS image. For example, there are some apparent differences in the connective stroma on the right side of the images. These differences in tissue morphology are expected as the PARS and H&E images are captured from tissue layers several microns apart.

In comparison with previous PARS-based emulated H&E imaging, the proposed method provides several benefits. Previous implementations developed by our group leverage a multiwavelength tunable excitation to capture hyperspectral images of several chromophores in the tissue.^17^^,^^18^ In contrast, the proposed technique requires only a single acquisition to be captured as the PARS absorption and scattering images are acquired concurrently. As a result, the scattering and absorption images are perfectly coregistered. Moreover, with bulk tissue contrast captured by the detection source, only UV excitation is required to capture nuclear structures. Thus, a costly and slow tunable excitation laser is no longer required. Instead, one of the many widely available 266-nm sources can be used for the PARS excitation. In this implementation, we use a 50-kHz UV excitation, which provides emulated H&E images substantially faster than the 1-kHz tunable source used in previous studies.^17^^,^^18^ For example, the image presented in Fig. 5 is captured in under 15 min with the proposed dual-contrast system, whereas it would require nearly 13 h to capture with the previously reported tunable system. The primary consideration with this technique then is the detection source. In this case, the 1310-nm detection is selected to leverage the disparity in the optical scattering properties of the preserved tissue and the surrounding substrate. Based on this difference, we may visualize tissue structures. However, the provided contrast is relatively limited. Optical scattering does not allow the high sensitivity and specificity provided by the 420-nm absorption contrast of cytochromes. The optical scattering visualizations do not provide the specificity for chromophore differentiation. Moreover, the scattering images may suffer from interference artifacts introduced by the lack of specificity. For example, deformities in the surface of the samples may also contribute to variations in optical scattering. For similar reasons, the current implementation does not perform well in freshly resected or formalin-fixed tissues. In these samples, there is no substrate present to provide a difference in scattering contrast. Furthermore, the 1310-nm detection lies within an optical window in tissue.^22^ Therefore, moving forward a shorter visible wavelength detection source may be implemented to provide better scattering contrast in a wider variety of samples. In addition, moving to a shorter detection wavelength will improve the ∼4.5-μm spatial resolution of the 1310-nm PARS scattering images. Ideally, if a detection wavelength is selected to target high optical scattering in tissues, this method could be applied in freshly resected or formalin-fixed tissue samples.

## 4 Conclusions

By isolating and leveraging the different contrast mechanisms provided by the PARS excitation and detection, we provide the first technique for single acquisition recovery of label-free emulated H&E images in preserved tissue blocks and sections. The combination of the two PARS contrast mechanisms provides rapid visualization of critical diagnostic features. While the PARS UV absorption captures nuclear contrast analogous to hematoxylin staining, the PARS scattering captures bulk tissue contrast analogous to eosin staining. Combining the two different images provides a direct emulation of the current gold standard for histopathological imaging. As presented here, these emulated H&E visualizations are provided directly on unstained FFPE blocks and in thin tissue sections. The images captured with this technique provide comparable contrast and resolution to the gold standard technique. Several salient features such as the nuclear organization, the connective tissue stroma, and the relationship between the highly nuclear cells to the connective tissue are important for deriving diagnostic information as the PARS H&E-simulated images highlight here. By visualizing nuclear and bulk tissue structure directly on the thick FFPE specimens, the proposed dual-contrast PARS microscope could potentially remove the need for sectioning and staining. This would expedite the histopathological workflow, saving time during the diagnostic process and removing potential for variability between successive tissue block sectioning for thin film slides. Moreover, PARS imaging is nondestructive and does not affect immunohistochemical staining efficacy. As a direct result, tissue blocks or unstained tissue samples could be imaged with PARS prior to undergoing further immunohistochemical processing, directly reducing premature consumption of small biopsy specimens. Overall, the label-free noncontact microscope presented here holds great potential as an adjunct to existing histopathological workflows. Adopted in a clinical setting, this microscope could provide a tabletop device for direct histological assessment of unstained embedded tissues.

## Code, Data, and Materials Availability

All data sets and materials relevant to this study are included in this article.
