# Single acquisition label-free histology-like imaging with dual-contrast photoacoustic remote sensing microscopy (Errata)

**DOI:** 10.1117/1.JBO.26.6.069801

**Published:** 2021-06-02

**Authors:** Benjamin Ecclestone, Deepak Dinakaran, Parsin Haji Reza

**Affiliations:** a University of Waterloo, Faculty of Engineering, Systems Design Engineering, PhotoMedicine Labs, Waterloo, Ontario, Canada; b illumiSonics, Waterloo, Canada; c University of Alberta, Department of Oncology, Edmonton, Alberta, Canada

## Abstract

The errata correct the errors in citation numbering that appeared in the originally published article.

This article [*J. Biomed. Opt.* **26**(5), 056007 (2021) doi: 10.1117/1.JBO.26.5.056007] was originally published on 25 May 2021 with erroneous citation numbering in the reference list and in the text.

In the reference list, the following references were corrected:

• from 12 to 13: G. Stasi and E.M. Ruoti “A critical evaluation in the delivery of the ultrasound practice: the point of view of the radiologist,” *Ital. J. Med.* **9**(1), 5 (2015)
• from 13 to 12: T. T. W. Wong et al., “Fast label-free multilayered histology-like imaging of human breast cancer by photoacoustic microscopy,” *Sci. Adv.* **3**(5), e1602168 (2017)
• from 17 to 19: N. J. M. Haven et al., “Ultraviolet photoacoustic remote sensing microscopy,” *Opt. Lett*. **44**, 3586–3589 (2019)
• from 18 to 20: N. Haven et al., “Reflective objective-based ultraviolet photoacoustic remote sensing virtual histopathology,” *Opt. Lett*. **45**, 535–538 (2020)
• from 19 to 17: K. Bell et al., “Reflection-mode virtual histology using photoacoustic remote sensing microscopy,” *Sci. Rep*. **10**, 19121 (2020)
• from 20 to 18: B.R. Ecclestone et al., “Improving maximal safe brain tumor resection with photoacoustic remote sensing microscopy,” *Sci. Rep*. **10**, 17211 (2020).

In the body of the text, the following citations were corrected:

• from 12 to 13, for the sentence
  ▪ “Acoustic transducers are typically bulky, have known interoperator technique reliability issues, and sometimes require immersion in a coupling media such as water to function.”
• from 13 to 14, for the sentences
  ▪ “PARS replaces the acoustically coupled ultrasound transducer with a detection laser.”
  ▪ “Photoacoustic signals are then detected as pressure-induced modulations in the backscattered magnitude of the detection beam.”
  ▪ “Observing backscattering in a reflection mode architecture allows PARS to image thick samples.”
• from 14 and 15, to 15-18, for the sentence
  ▪ “Moreover, PARS may provide chromophore-specific contrast by selecting excitation wavelengths to target unique biomolecule absorption spectra.”
• from 19 and 20, to 17 and 18, for the sentences
  ▪ “Previously, PARS has provided complete H&E emulation using a tunable excitation source to independently target the absorption peaks of DNA and cell membrane structures.”
  ▪ “While effective in both thin sections and tissue blocks, this technique was largely limited in field of view, resolution, and imaging speed since it required the use of a slow (1 kHz) tunable excitation source.”
• from 13 to 14, for the sentences
  ▪ “In PARS systems, a cofocused pulsed excitation and continuous wave detection laser pair are used to capture photoacoustic absorption contrast.”
  ▪ “The excitation induces photoacoustic signals by depositing focused pulses of optical energy into the sample.”
  ▪ “The absorption contrast is then captured as nanosecond scale pressure-induced modulations in the backscattered intensity of the cofocused detection laser.”
  ▪ “Usually, to capture MHz-scale PARS modulations, the time-resolved backscattering magnitude is band pass filtered to isolate the absorption signal.”
• from 16 and 17, to 17 and 18 for the sentences
  ▪ “Previous implementations developed by our group leverage a multiwavelength tunable excitation to capture hyperspectral images of several chromophores in the tissue.”
  ▪ “In this implementation, we use a 50-kHz UVexcitation, which provides emulated H&E images substantially faster than the 1-kHz tunable source used in previous studies.”

The above-listed errors were corrected, and the article was republished on 26 May 2021.

